# SOX30 is a key regulator of desmosomal gene suppressing tumor growth and metastasis in lung adenocarcinoma

**DOI:** 10.1186/s13046-018-0778-3

**Published:** 2018-05-31

**Authors:** Xianglin Hao, Fei Han, Bangjin Ma, Ning Zhang, Hongqiang Chen, Xiao Jiang, Li Yin, Wenbin Liu, Lin Ao, Jia Cao, Jinyi Liu

**Affiliations:** Institute of Toxicology, College of Preventive Medicine, Third Military Medical University, 30 Gaotanyan Street, Shapingba District, Chongqing, 400038 People’s Republic of China

**Keywords:** SOX30, Desmosome, Wnt, ERK, Lung adenocarcinoma

## Abstract

**Background:**

The expression of desmosomal genes in lung adenocarcinoma and lung squamous carcinoma is different. However, the regulatory mechanism of desmosomal gene expression in lung adenocarcinoma and lung squamous carcinoma remains unknown.

**Methods:**

The correlation between expression of desmosomal gene expression and SOX30 expression were analyzed by bioinformatics. The expression of SOX30, DSP, JUP and DSC3 were detected in lung cancer cell lines, lung tissues of mice and patients’ tissues by qPCR, WB, Immunofluorescence and Immunohistochemistry. A chromatin Immunoprecipitation assay was used to investigate the mechanisms of the SOX30 regulation on desmosomal gene expression. In vitro proliferation, migration and invasion assays, and an in vivo nude mice model were utilized to assess the important role of desmosomal genes on SOX30-induced tumor suppression. A WB assay and TOP/FOP flash reporter assay was used to investigate the downstream pathway regulated by the SOX30-desmosomal gene axis. A chemical carcinogenic model of SOX30-knockout mice was generated to confirm the role of the SOX30-desmosomal gene axis in tumorigenesis.

**Results:**

The expression of desmosomal genes were upregulated by SOX30 in lung adenocarcinoma but not in lung squamous carcinoma. Further mechanism studies showed that SOX30 acts as a key transcriptional regulator of desmosomal genes by directly binding to the ACAAT motif of desmosomal genes promoter region and activating their transcription in lung adenocarcinoma. Knockdown of the expression of related desmosomal genes by miRNA significantly attenuated the inhibitory effect of SOX30 on cell proliferation, migration and invasion in vitro and on tumor growth and metastasis in vivo. In addition, knockout of SOX30 promotes lung tumor development and loss the inhibition of desmosomal genes on downstream Wnt and ERK signal in urethane-induced lung carcinogenesis in SOX30-knockout mice.

**Conclusions:**

Overall, these findings demonstrate for the first time that SOX30 acts as a master switch of desmosomal genes, inhibits lung adenocarcinoma cell proliferation, migration and invasion by activating the transcription of desmosomal genes. This study provides novel insights on the regulatory mechanism of desmosomal genes in lung adenocarcinoma.

**Electronic supplementary material:**

The online version of this article (10.1186/s13046-018-0778-3) contains supplementary material, which is available to authorized users.

## Background

Lung cancer is the leading cause of cancer-related incidence and mortality throughout the world [1]. Non-small cell lung cancer (NSCLC) is the major subtype of lung cancer, which is typically divided into two histological subtypes, lung adenocarcinoma (ADC) and lung squamous carcinoma (SCC) [2]. In addition to differences in morphology, the underlying mechanisms, molecular profiling and therapeutic methods are quite different [3–5]. Therefore, identifying differentially-expressed genes between ADC and SCC is useful to better understand their pathogenesis.

In the process of cancer development, cell junction molecules play a critical role in inhibiting tumor growth and metastasis [6–10]. Previous evidence revealed that desmosomal proteins, as a member of cell junction molecules, are deregulated in various cancers, including lung cancer [11]. In lung cancer, different members of desmosome genes have different roles in tumor progression. For example, plakophilin 1 (PKP1) can be useful in the diagnosis of patients with SCC [12, 13]. Plakophilin 3 (PKP3) promotes tumor growth in lung cancer [14]. Decreased desmocollin 1 (DSC1) expression is associated with poor prognosis in human lung cancer [15]. Desmoglein 2 (DSG2) acts as an oncogene by accelerating tumor growth of non-small-cell lung carcinoma (NSCLC) [16]. Desmoglein 3 (DSG3) is underexpressed in lung cancer, and a lower expression of DSG3 is significantly linked to higher tumor grade [17]. However, the downstream signaling pathway of these desmosomal genes in regulating tumor progression is not entirely clear, except for the three canonical tumor suppressor desmoplakin (DSP), junction plakoglobin (JUP) and desmocollin 3 (DSC3). The expression of DSP and JUP are reduced or absent in lung cancer and re-expression results in reduction of cell growth and migration by suppressing the Wnt signaling pathway in lung cancer [12–14]. DSC3 acts as a tumor suppressor through inhibiting the EGFR/ERK pathway in lung cancer [17, 18]. In previous studies, higher expression of desmosomal genes were found in SCC tissues compared with ADC tissues [2, 4, 17, 19, 20]. However, the differentce in regulatory mechanisms of desmosomal genes between ADC and SCC are still not clear.

SOX30 is a member of the Sox family of transcription factors that has been isolated from Fugu, human, mouse and the Nile tilapia [21]. A high SOX30 expression has a favorable and independent prognostic factor for ADC patients but not for SCC patients [22]. Our in vitro experiments show that SOX30 overexpression significantly inhibits proliferation by inducing apoptosis in ADC but not in SCC [19, 22]. These studies indicate that SOX30 may have different functional roles in distinct subtypes of lung cancer. Thus, the different downstream signal mechanism of SOX30 in these two histological subtypes of lung cancer needs to be further investigated.

In this study, our data demonstrates that the expressions of desmosomal genes are regulated by SOX30 in ADC but not in SCC. Mechanistically, SOX30 activates desmosomal gene expression through direct binding to the promoter regions of the desmosomal genes in ADC cells. Our findings elucidate the regulatory mechanisms of desmosomal genes in ADC.

## Methods

### Cell lines

The lung cancer cell lines (A549, LTEP-a-2, H520 and H226) were obtained from the Cell Bank of the Chinese Academy of Science (Shanghai, China), cultured in RPMI-1640 medium supplemented with 10% fetal bovine serum (Gibco, CA). All the cells were maintained at 37 °C with 5% CO_2_.

### Plasmid construction and cell transfection

Construction of SOX30 expression vector was performed as previously described [19, 22]. For knockdown, two pairs of oligomeric singlestranded oligonucleotides and a pair of negative oligomeric singlestranded oligonucleotides were synthesized, and then inserted into miRNA expression vector pcDNA6.2-GW/EmGFP-miR. Cells were transfected using Lipofectamine2000 Reagent (Invitrogen Preservation, Carlsbad, CA, USA) according to the manufacturer’s instructions. The stably transfected cells were screened under G418 (Calbiochem, La Jolla, CA, USA) or Blasticidin (Sigma). Cell clones were obtained by the limited diluted method.

### RT-PCR and qRT–PCR analysis

The RNA of cells was isolated using the Trizol reagent (Invitrogen, Life Technologies), and conversion of total RNA to cDNA was performed with the Reverse Transcription System (Promega, Madison, WI, USA). All qRT-PCR reactions was performed using the C1000 Real-Time Cycler (Bio-Rad Laboratories, Hercules, CA, USA) and qRT-PCR Master mixes (Promega, Madison, WI, USA). Primers for amplification of the DSC1, DSG1, DSC2, JUP, DSP, PKP1, DSC3, DSG3, PKP3 and ACTIN genes are listed in Additional file 1: Table S1. All experiments were carried out in triplicate, and the 2^-ΔΔt^ method was used to determine expression of the genes of interest.

### MTS

For knockdown, A549 and LTEP-a-2 cells with SOX30 or empty vector stably expression were plated at 4 × 10^3^ cells per well on 96-well plates, and transfected with desmosomal gene miRNA or negative control. Cell proliferation was assessed using MTS Reagent (Promega, Madison, WI, USA) on days 1, 2, 3, 4 and 5 after transfection. The assays were performed in triplicate.

### Colony formation assay

Stable transfected A549 and LTEP-a-2 cells (*n* = 1500) were seeded into 100 mm cell culture dish and maintained in media for 14 days. Surviving colonies were fixed with 4% paraformaldehyde and stained with 0.1% crystal violet (Beyotime Biotechnology, China) for 15 min. Then, the cell colonies were eluted by acetic acid and the absorbance of crystal violet were measured at 570 nm. The experiment was carried out in triplicate wells for three times.

### Boyden chamber migration/invasion assay

Transwell assays were performed by using transwell plates with 8 μm pore (Corning; 3422). For the migration assay, 1 × 10^4^ Cells were resuspended in serum-free RPMI-1640 medium and plated onto the 24-well upper chamber with the lower chamber filled with complete medium. For the invasion assay, the 24 transwell plate with matrigel (BD biosciences) polymerized at the 24-well upper chamber for 2 h at 37 °C as the intervening invasive barrier. After 24 h incubation at 37 °C, the cells on the upper chamber were removed and the number of cells that migrated or invaded to the lower side were fixed in 4% paraformaldehyde and stained with 1% crystal violet and counted at × 200 magnification in 10 different fields of microscope (Leica; D-35578). The results were determined from three repeated experiments.

### In vivo tumorigenicity assay

For the xenograft tumor growth assay, a total of 1 × 10^6^ stable transfected A549 cells suspended in 150 μl PBS were injected subcutaneously into the right flanks of 5-week-old male Balb/c nude mice (*n* = 4 mice per group), respectively. Tumors size were measured every 3–5 days with calipers after injection, and the tumor volume was calculated based on formula: 0.5 × (length × width^2^). After 42 days housing, the mice implanted tumors were sacrificed, and liver tissues were dissected and subjected to histological examination. Metastatic nodules were detected by H&E staining and quantified by counting metastatic lesions in each section. The images of the positive areas were taken. All experimental animal procedures were approved by the Institutional Animal Care and Use Committee of Third Military Medical University, China.

### WB analysis

WB was performed as previously described [20]. ACTIN was used as a loading control. The following primary antibodies were used: SOX30 rabbit polyclonal antibody (1:100; Santa Cruz Biotechnology; sc-20,104), DSP rabbit polyclonal antibody (1:100; Santa Cruz Biotechnology; sc-33,586), JUP rabbit polyclonal antibody (1:100; Santa Cruz Biotechnology; sc-7900), DSC3 rabbit polyclonal antibody (1:100; Santa Cruz Biotechnology; sc-48,750), CCND1 rabbit polyclonal antibody (1:100; Santa Cruz Biotechnology; sc-717), MMP7 rabbit polyclonal antibody (1:100; Santa Cruz Biotechnology; sc-30,071), ERK1/2 rabbit polyclonal antibody (1:100; Santa Cruz Biotechnology; sc-93), p-ERK1/2 rabbit polyclonal antibody (1:100; Santa Cruz Biotechnology; sc-101,761) and ACTIN monoclonal antibody (1:2000; Sigma; A5441). ImageJ software was used to quantify the protein expression.

### Immunofluorescence cell staining (IF)

Lung cancer cell lines with SOX30 or vector control stable expression grown on sterile glass coverslips, washed with PBS, fixed in 4% paraformaldehyde for 15 min and permeabilized by 0.5% Triton for 15 min at room temperature. Following blocking with 1% bovine serum albumin for 30 min, rabbit polyclonal antibody SOX30 (1:100; Santa Cruz Biotechnology; sc-20,104), mouse monoclonal antibody DSP (1:100; Santa Cruz Biotechnology; sc-365,981), mouse monoclonal antibody JUP (1:100; Santa Cruz Biotechnology; sc-33,634) and mouse monoclonal antibody DSC3 (1:100; Santa Cruz Biotechnology; sc-81,806) were incubated overnight at 4 °C. After washing three times, the cells were incubated with an appropriate fluorochrome-conjugated secondary antibody (1:300; Invitrogen; A-11010 and A-11003) for 1 h at 37 °C in the dark. After blue nuclear counterstaining with 4, 6-diamidino-2-phenylindole (Beyotime, Shanghai, China; C1006) for 10 min at room temperature, coverslips were mounted and observed with a fluorescence microscope (Zeiss, Oberkochen, Germany; LSM800). ZEN software was used to quantify the protein expression.

### Patient sample and immunohistochemical (IHC) analysis

Tissue microarrays contained a total of 30 lung cancer patient tissue samples including 15 ADCs and 15 SCCs were obtained from the collaboration (Shanghai Biochip Co Ltd., Shanghai, People’s Republic of China). The rabbit polyclonal antibodies used were SOX30 rabbit polyclonal antibody (1:100; Santa Cruz Biotechnology; sc-20,104), DSP rabbit polyclonal antibody (1:100; Santa Cruz Biotechnology; sc-33,586), JUP rabbit polyclonal antibody (1:100; Santa Cruz Biotechnology; sc-7900), DSC3 rabbit polyclonal antibody (1:100; Santa Cruz Biotechnology; sc-48,750). IHC staining was performed as described previously [19].

### Chromatin immunoprecipitation (ChIP) assay

ChIP analysis was performed using a ChIP Assay Kit (Pierce, Rockford, IL, USA). The immunoprecipitated and input DNA was used as a template for RT–PCR analysis using the primers listed in Additional file 1: Table S1.

### Chemoncogenic model

C57BL/6 J (SOX30 conditional knockout) mice were obtained from the model animal research center of Nanjing University (Nanjing, China) and maintained in a controlled environment (12-h light/dark cycle, ad libitum access to food and water) at the laboratory animal facility of Third Military Medical University (Chongqing, China). The *W*/W mice (*n* = 5) and K/K mice (*n* = 5) that were used were matched for age (14 weeks) and weight (16–19 g). These mice received a intraperitoneal injection of urethane (1 g/kg in 100 μl saline) once a week for 10 consecutive weeks. Mice were sacrificed after 4 months. Lung neoplastic lesions were observed by H&E staining. All experiments on mice were approved by the Institutional Animal Care and Use Committee of Third Military Medical University, China.

### Analysis of publicly available datasets

GSE43580 dataset were used to analyze differential gene expression between ADC and SCC. The protein expression of desmosomal genes in human lung tumor tissues were determined from the human protein atlas (www.proteinatlas.org). TCGA lung adenocarcinoma RNAseq data (*n* = 571) and TCGA lung squamous carcinoma RNAseq data (*n* = 553) were used to compare the expression of SOX30 with desmosomal genes.

### Statistical analysis

Statistical analyses were performed with the SPSS 16.0 software (SPSS, Inc., Chicago, IL, USA). Each experiment was performed at least three times. The data were presented as the means ± SD. Results of expression analyses, cell proliferation, colony formation, migration and invasion were analyzed using the two-tailed Student’s t-test. Correlation analysis of gene expression was performed using Spearman’s rank correlation coefficient analysis. A two-sided *P*-value<0.05 was taken as statistically significant.

## Results

### Identification of differentially expressed genes between ADC and SCC

**Fig. 1:**
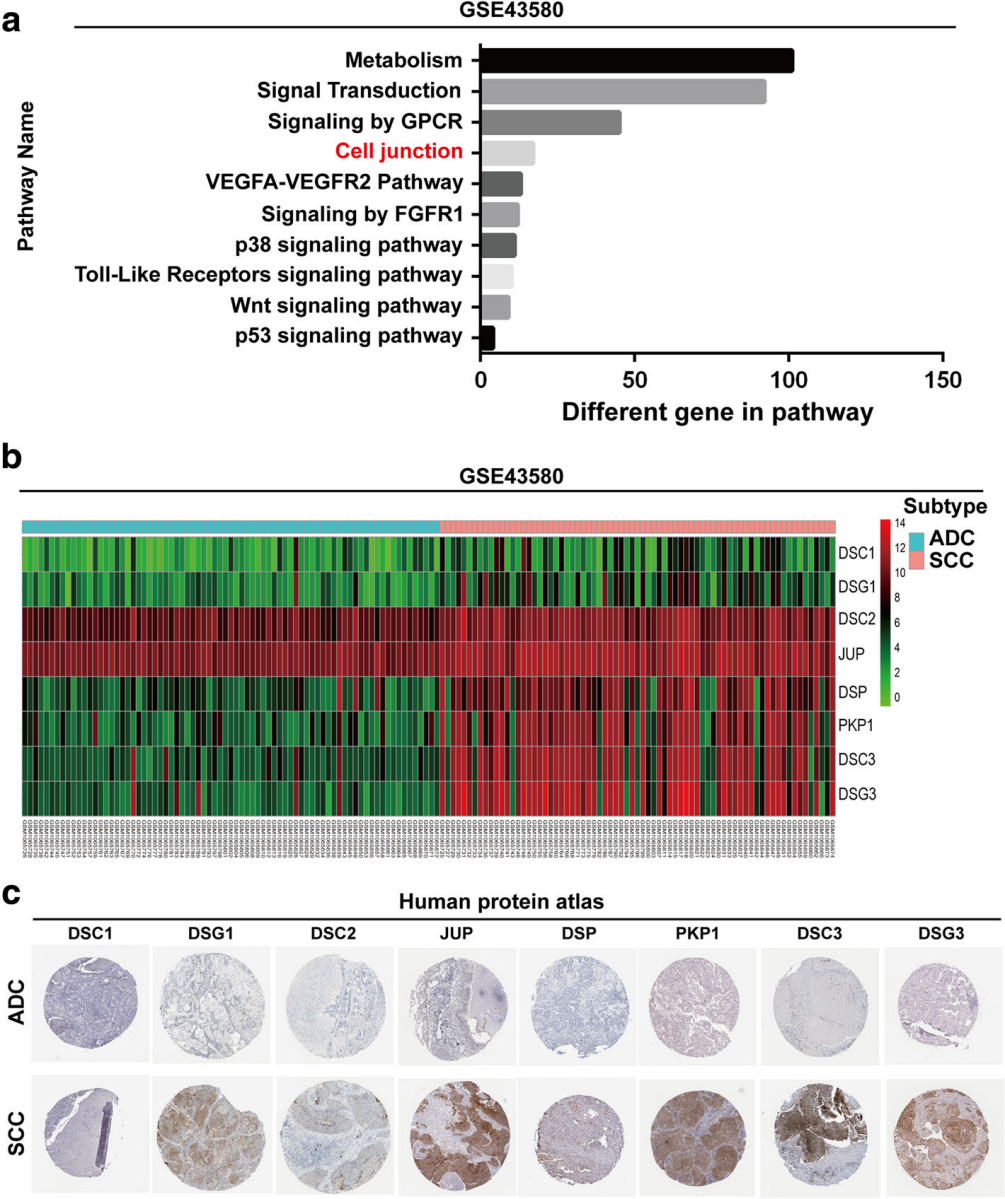
The expressions of desmosomal genes are different between ADC and SCC. **a** Identification of differential genes between ADC and SCC with GSE-43580 dataset. **b** Analysis of desmosomal gene expression levels between ADC and SCC using GSE43580 dataset. **c** The expression of desmosomal genes in ADC specimens and SCC specimens. Images were taken from the Human Protein Atlas (http://www.proteinatlas.org) online database

To analyze the differential gene expression between ADC and SCC, we first performed pathway analysis of one large-sample lung cancer datasets-GSE43580 and found that the expression of 17 cell junction genes has significant differences between ADC and SCC (Fig. 1a). Gene ontology (GO) analysis was conducted based on the 17 genes identified in the pathway analysis revealing 8 desmosomal genes: DSC1, DSG1, DSC2, JUP, DSP, PKP1, DSC3 and DSG3 (Fig. 1b). To further confirm the GO results, we analyzed the protein expression of these 8 desmosomal genes in clinical specimens from the human protein atlas (www.proteinatlas.org). We found that all desmosomal genes were overexpressed in SCC, and underexpressed in ADC (Fig. 1c and Table 1).

**Table 1:** Positive percentage of desmosomal gene expression between ADC and SCC

Genename	Desmosomal gene expression									
	ADC				PR(%)	SCC				PR(%)
	HIGH	MID	LOW	ND		HIGH	MID	LOW	ND	
DSC1	0	0	2	10	16.7	0	4	3	2	77.8
DSG1	0	0	8	16	33.3	0	5	6	5	68.8
DSC2	0	18	4	2	91.7	0	19	4	0	100
JUP	9	4	4	7	70.8	9	5	2	1	94.1
DSP	0	0	4	19	17.4	0	0	2	8	20.0
PKP1	0	0	0	16	0.0	7	4	2	3	81.3
DSC3	0	0	4	18	18.2	2	6	3	4	73.3
DSG3	0	0	2	20	9.1	0	4	0	16	20.0

*HIGH* High expression, *MID* Middle expression, *LOW* Low expression, *ND* Not detected, *PR (%)* positive rate

### Desmosomal gene expression positively correlates with SOX30 expression in ADC

**Fig. 2:**
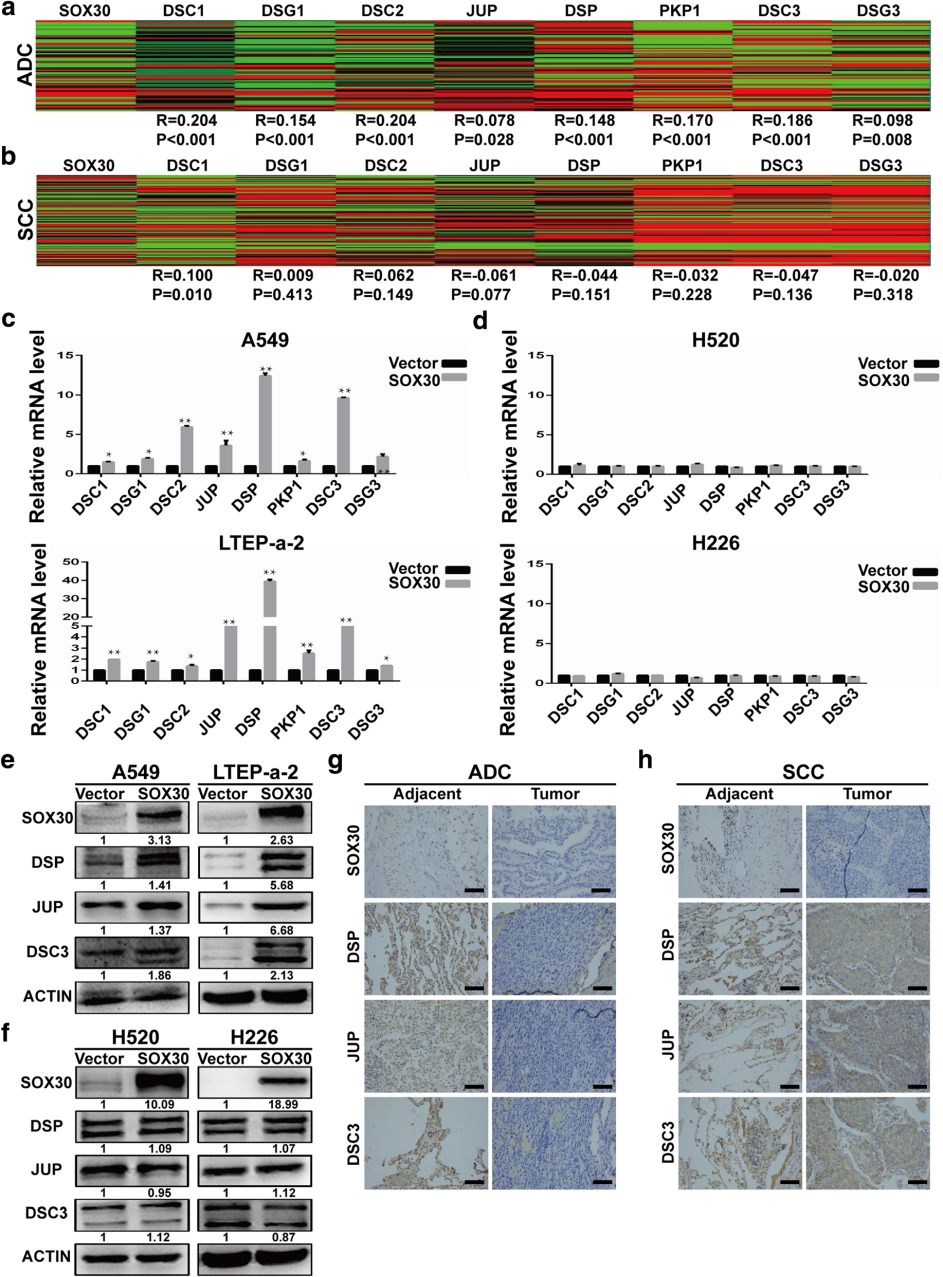
SOX30 is positive correlated with desmosomal gene expression in ADC, but not in SCC. **a** Heatmaps for correlations between SOX30 and desmosomal genes in the TCGA lung adenocarcinoma RNAseq (IlluminaHiSeq; *n* = 571) data set. **b** Heatmaps for correlations between SOX30 and desmosomal genes in the TCGA lung squamous carcinoma RNAseq (IlluminaHiSeq; *n* = 553) data set. Correlation coefficient R and *P*-values were calculated by a Spearman correlation analysis. **c** qRT-PCR analysis of desmosomal gene expression in A549 and LTEP-a-2 cells transiently transfected with the vector control or SOX30 expression vector. **d** qRT-PCR analysis of desmosomal gene expression in H520 and H226 cells transiently transfected with the vector control or SOX30 expression vector. ACTIN was used as an internal control. **e** The protein levels of DSP, and JUP and DSC3 were monitored by WB after SOX30 overexpression in A549 and LTEP-a-2 cells. **f** The protein levels of DSP, and JUP and DSC3 were monitored by WB after SOX30 overexpression in H520 and H226 cells. **g** The protein levels of SOX30, DSP, JUP and DSC3 were further monitored by IHC in human ADC tissues. **h** The protein levels of SOX30, DSP, JUP and DSC3 were further monitored by IHC in human SCC tissues. Scale bar represents 50 mm

Our previous research suggested that SOX30 is a tumor suppressor in ADC but not in SCC [19, 22]. Thus, we suspect that the expression of desmosomal genes are associated with SOX30 expression. Therefore, we analyzed the correlation of SOX30 expression with the 8 desmosomal genes identified in the previous experiments between ADC and SCC by using TCGA data. The results revealed that the expressions of desmosomal genes were significantly positively correlated with SOX30 expression in ADC but not in SCC (Fig. 2a and b). Previous studies revealed that desmosome family members play a crucial role in tumor suppression [21]. Therefore, we hypothesized that SOX30 may inhibit tumor progression through upregulation of the desmosomal gene expression in ADC but not in SCC. To examine this hypothesis, qRT-PCR analyses were performed to measure the expression of desmosomal genes in SOX30-transfected A549 and LTEP-a-2 cells (two human lung adenocarcinoma cell lines), and H520 and H226 cells (two human lung squamous carcinoma cell lines). Consistently, the overexpression of SOX30 upregulated the expressions of the 8 desmosomal genes in the A549 and LTEP-a-2 cells but not in the H520 and H226 cells (Fig. 2c and d). Among these 8 desmosomal genes, the most obvious change in expression is three tumor suppressor genes for lung cancer, DSP, JUP and DSC3. To identify the functional target gene of SOX30, we determined the protein expression of these desmosomal genes by WB analyses and immunofluorescence cell staining. The results showed that these genes were upregulated in the SOX30 high expression group of A549 and LTEP-a-2 cells but not in the H520 and H226 cells (Fig. 2e and f, Additional file 2: Figure S1). To further determine the correction between SOX30 and these genes, we tested for expression of SOX30, DSP, JUP and DSC3 in human lung cancer tissues and adjacent tissues. The expression of DSP, JUP and DSC3 were associated with SOX30 expression in human ADC tissues but not in human SCC tissues (Fig. 2g and h).

### SOX30 upregulates the expression of DSP, JUP and DSC3 by directly binding to their promoter region in lung adenocarcinoma cells

**Fig. 3:**
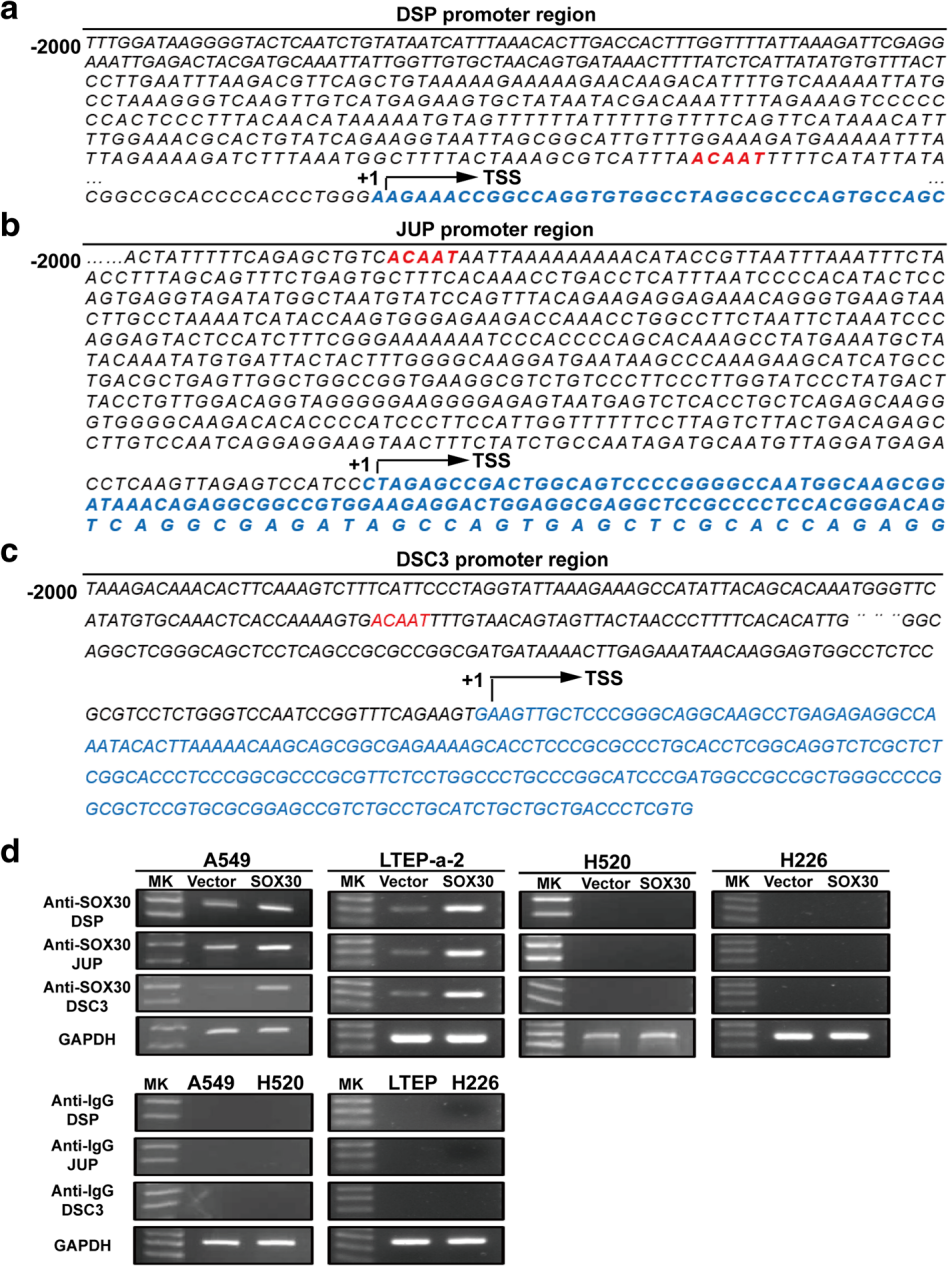
SOX30 directly binding to promoters of DSP, JUP and DSC3. **a–c** The ACAAT motif in promoter region of DSP, JUP and DSC3 were underlined. TSS, transcription start site. **d** ChIP-PCR was performed to identify DSP, JUP and DSC3 are direct binding targets of SOX30

Emiko et al. reported that SOX30 was able to specifically recognize the promoter of genes that have an ACAAT motif [23]. Furthermore, SOX30 can transcriptionally activate the target gene by directly binding to the promoter containing the ACAAT motif ref. Subsequently, analyzing the promoter region of DSP, JUP and DSC3, and found their promoter regions all contain an ACAAT motif (Fig. 3a–c). Then, we determined whether SOX30 could activate DSP, JUP and DSC3 transcription by binding to the ACAAT motif on their promoters. Chromatin immunoprecipitation (ChIP) assay demonstrated that SOX30 could directly bound to their promoters in A549 and LTEP-a-2 cells but not in H520 and H226 cells (Fig. 3d).

### DSP, JUP and DSC3 induction are critical for SOX30-mediated cell proliferation, migration and invasion in ADC cells in vitro

**Fig. 4:**
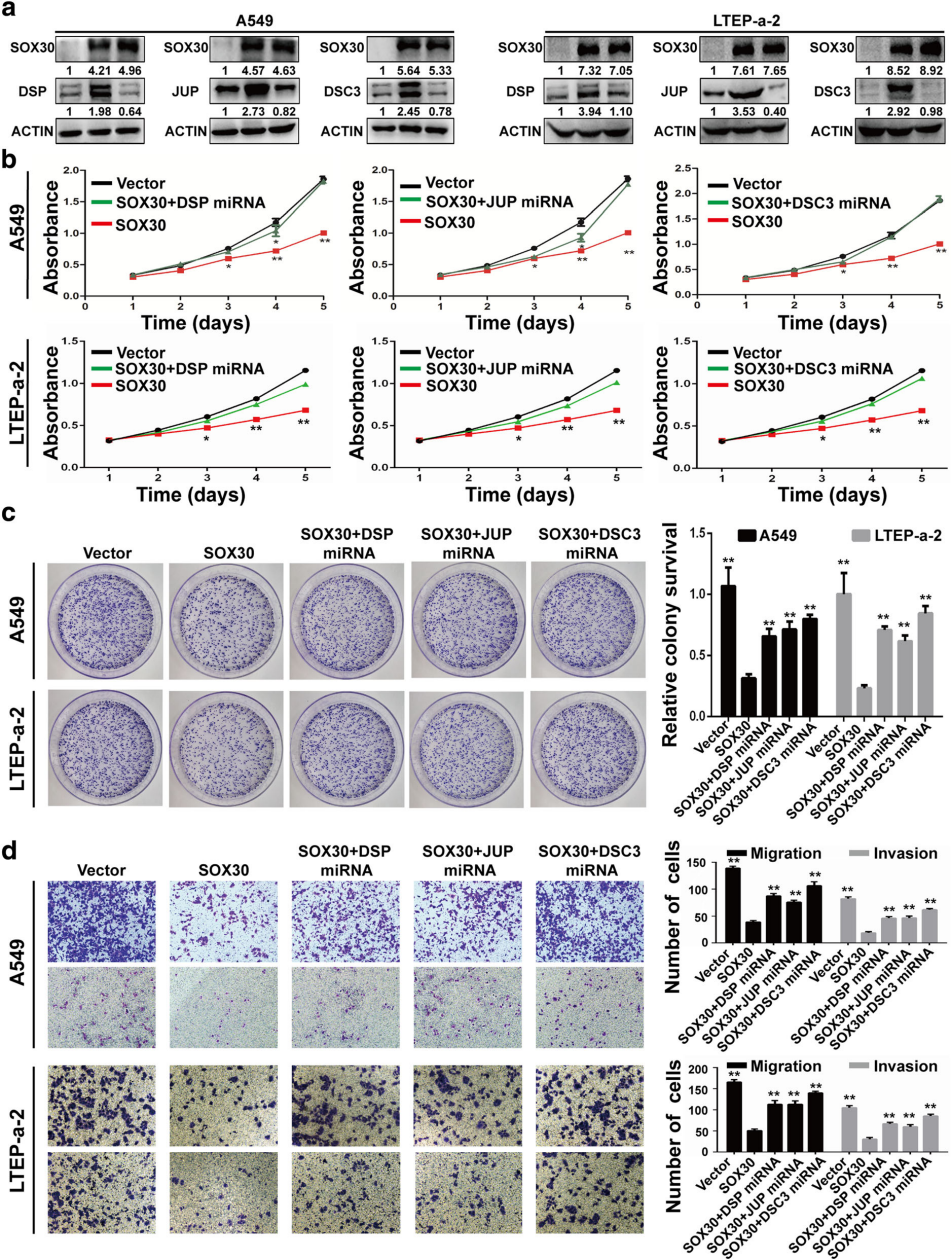
Desmosomal gene silencing diminished the effect of SOX30 overexpression on proliferation, migration and invasion of A549 and LTEP-a-2 cells. **a** SOX30, DSP, JUP and DSC3 expression were confirmed by WB in A549 and LTEP-a-2 cells. **b** MTS assays were performed to analyze cell proliferation of A549 and LTEP-a-2 cells cotransfected with desmosomal gene miRNA and SOX30 expression vector. *P < 0.05; **P < 0.01. **c** The effect of increasing SOX30 expression with decreasing desmosomal gene expression on cell growth was further confirmed by colony formation assay in A549 and LTEP-a-2 cells. **d** Assessment of the effects of the expression of SOX30 and desmosomal gene, respectively, on cell migration and invasion in A549 and LTEP-a-2 cells

To define whether DSP, JUP and DSC3 mediates the effect of SOX30 on cell proliferation, migration and invasion in ADC cells, we first constructed two SOX30-overexpressing ADC cell lines, (A549-SOX30(+)) and (LTEP-a-2-SOX30(+)), and their negative controls (A549-Vector) and (LTEP-a-2-Vector). We then blocked either DSP, JUP or DSC3 expression in A549-SOX30(+) and (LTEP-a-2-SOX30(+)) cells by using miRNA; thus establishing ADC cell lines that are SOX30-overexpressing with either DSP, JUP or DSC3 knockdown. The expression of SOX30, DSP, JUP and DSC3 were verified by WB analysis (Fig. 4a). The MTS, colony formation assay revealed that the knockdown of DSP, JUP or DSC3 by miRNA significantly abrogated the effects on cell proliferation induced by SOX30 overexpression in A549 and LTEP-a-2 cells (Fig. 4b–c). The transwell assay was performed to determine whether desmosomal genes are involved in SOX30-mediated migration and invasion of ADC cells. The results showed that interference with the expression of DSP, JUP or DSC3 reversed the inhibitory effect of SOX30 on cell migration and invasion (Fig. 4d).

### DSP, JUP and DSC3 are involved in SOX30-mediated tumor suppression of ADC in vivo

**Fig. 5:**
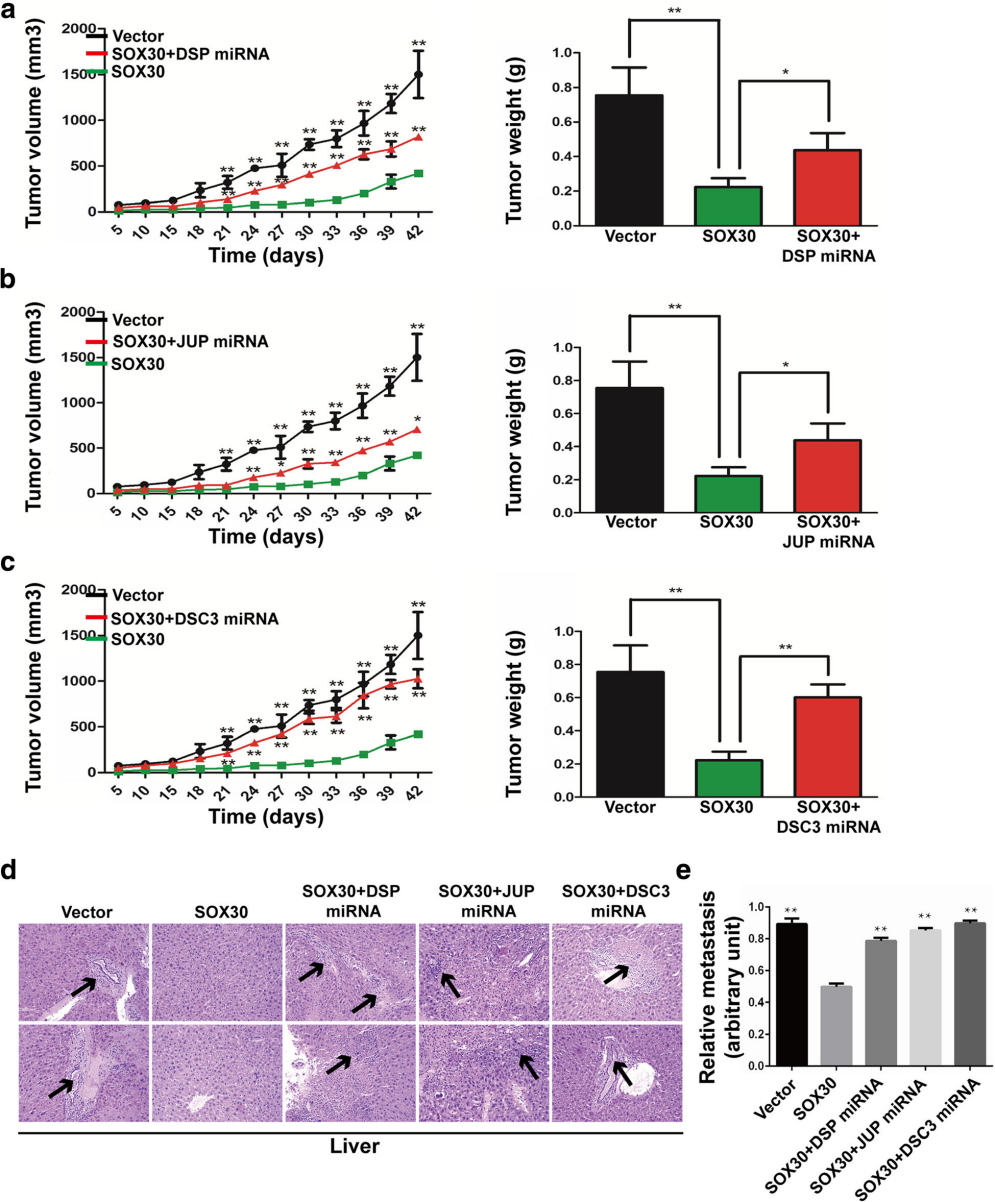
Knockdown of desmosomal gene expression attenuates the effects of SOX30 on impeding tumor formation and metastasis in vivo. **a**–**c** Evaluation of tumorigenesis in nude mice subcutaneously injected with (A549-Vector), (A549-SOX30(+)), (A549-SOX30(+)/DSP(−)), (A549-SOX30(+)/JUP(−)) and (A549-SOX30(+)/DSC3(−)). To assess the effects of desmosomal gene on subcutaneous tumor growth, tumor volumes were recorded on the indicated days, and pictures of solid tumor tissues were taken after 42 days. Tumor weights in the indicated groups were determined. Error bars indicate s.d. (*n* = 4). **d** Liver metastases were observed by H&E staining in the indicated groups. Arrows indicate the metastatic loci. **e** Liver metastasis was further quantified using RT-qPCR. Human-specific β2-MG levels were used to quantify metastatic human cancer cells with the mouse-specific β2-MG level as an internal control. Error bars indicate s.d. (n = 4).*P < 0.05; **P < 0.01

To further investigate whether upregulation of DSP, JUP and DSC3 are necessary for SOX30-medicated tumorigenesis and metastasis in vivo, we utilized a xenograft tumor model using these stable cell lines. As showed in Fig. 5a–c, xenografted (A549-SOX30(+)/DSP(−)), (A549-SOX30(+)/JUP(−)) and (A549-SOX30(+)/DSC3(−)) cells rapidly proliferated in mice compared with (A549-SOX30(+)) cells, and we found that tumor volume of (A549-SOX30(+)/DSP(−)), (A549-SOX30(+)/JUP(−)) and (A549-SOX30(+)/DSC3(−)) were significantly larger than those with (A549-SOX30(+)) cells. Moreover, H&E staining revealed that knockdown of DSP, JUP or DSC3 dramatically increased the number of metastatic foci in the liver of nude mice (Fig. 5d), and these results were further confirmed by qRT-PCR using a human-specific β2-MG (beta-2-microglobulin) with a mouse-specific β2-MG as an internal control (Fig. 5e) [24]. These results indicate that DSP, JUP and DSC3 play critical roles in SOX30-mediated growth and metastasis of ADC.

### SOX30 expression impairs Wnt signaling and ERK signaling through induction of desmosomal gene expression

**Fig. 6:**
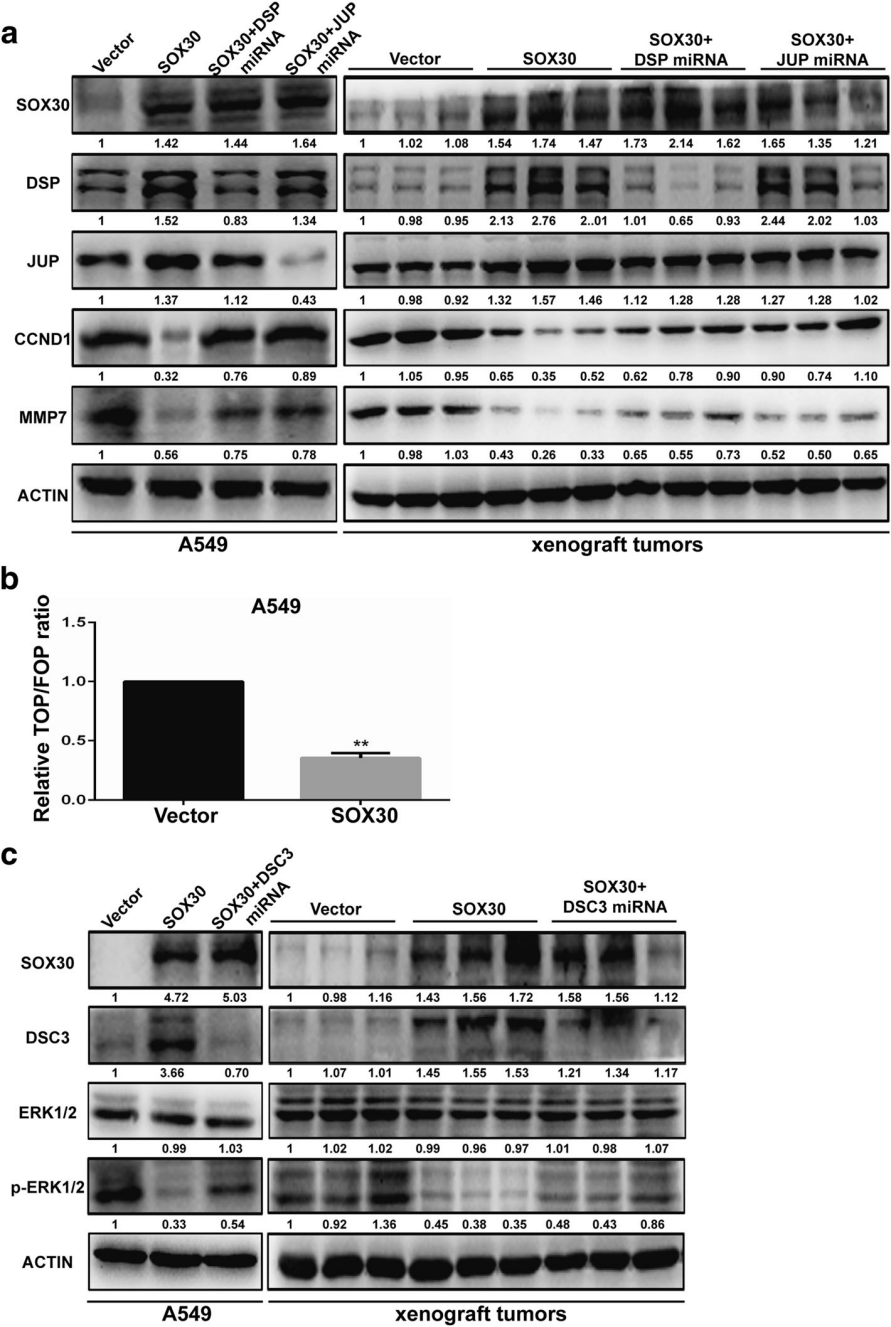
SOX30 inhibits Wnt and ERK signaling by accelerating the expression of desmosomal gene. **a** The protein expression of SOX30, DSP, JUP, CCND1 and MMP7 were detected in A549-SOX30 cells and subcutaneous tumors of mice. **b** A549 cells with SOX30 or vector control stable expression were co-transfected with luciferase reporter constructs (TOPFlash or FOPFlash, respectively) and Renilla luciferase constructs (normalizing transfection control). The reporter activity was measured 24 h after transfection. Error bars represent the s.d. of three independent experiments. ** SOX30 vs vector control, Student’s t-test, *P* < 0.01. **c** The protein expression of SOX30, DSC3, ERK1/2 and p-ERK1/2 were detected in A549-SOX30 cells and subcutaneous tumors of mice

**Fig. 7:**
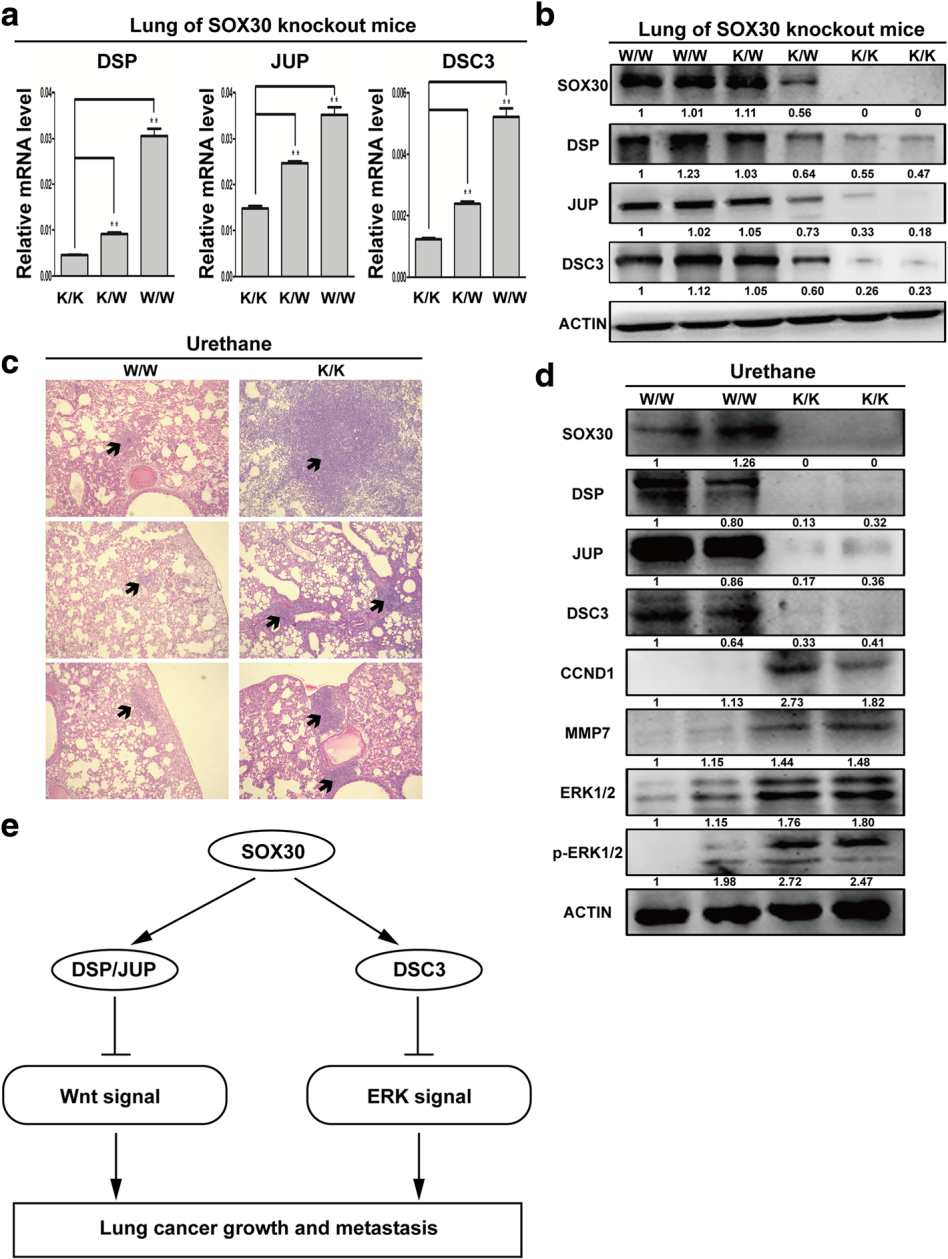
Inhibition of SOX30 promotes tumorigenesis of lung cancer with urethane treatment. **a** The mRNA expression of DSP, JUP and DSC3 in lung tissues of SOX30-knockout mice were measured by qRT-PCR. **b** The protein expression of DSP, JUP and DSC3 in lung tissues of SOX30-knockout mice were detected by WB. **c** Lung tissues were processed and stained with H&E for detection of tumor foci. **d** Tumor extracts were analyzed by WB. ACTIN was used as a loading control. **e** Schematic diagram of the mechanisms of SOX30 mediated suppression of ADC cell proliferation and metastasis based on our study

Previous research has shown that a member of SOX family mediates the Wnt signaling pathway [25–27]. According to our observation presented above and considering the facts that DSP and JUP can suppress lung cancer progression by inhibition of the Wnt/β-catenin signaling pathway, we hypothesized that SOX30 can suppress the Wnt/β-catenin signaling pathway through upregulation of DSP and JUP. To test this hypothesis, the protein expression levels of SOX30, DSP, JUP, MMP7 and CCND1 in A549 stable cell lines and xenograft tumors were first examined by WB. Consistent with our theory, the protein expression of MMP7 and CCND1 were downregulated in a SOX30-overexpressing cell line and xenograft tumors; MMP7 and CCND1 expression were increased significantly in SOX30-overexpressing cells, or tumor tissue after DSP or JUP expression was blocked (Fig. 6a). We then conducted a TOP/FOP FLASH reporter assay in SOX30-overexpressing A549 cells. As shown in Fig. 7a, overexpression of SOX30 significantly inhibited the transcription activity of β-catenin/T-cell factor (TCF) compared with cells transfected with the vector control (Fig. 6b). Previous studies have reported that DSC3 acts as a tumor suppressor through inhibiting ERK signaling in human lung cancer [28]. WB assay were performed to test whether these changes affect ERK1/2-related signaling activity in SOX30-transfected A549 cells. The data showed that overexpression of SOX30 significantly reduced the phosphorylation of ERK1/2 whereas the knockdown of DSC3 significantly abrogated the SOX30 overexpression-induced dephosphorylation of ERK1/2, suggesting that DSC3 is involved in the SOX30-regulated dephosphorylation of ERK1/2 in vitro and in vivo (Fig. 6c). Therefore, these results indicated that SOX30 overexpression suppresses Wnt/β-catenin signaling and ERK signaling by increasing the expression levels of desmosomal genes, and this may contribute to inhibition of cell growth, migration and invasion in ADC.

### The accelerating urethane-induced lung tumorigenesis of SOX30 loss is associated with desmosomal genes

To further define the important role of the “SOX30-desmosomal gene axis” axis in lung carcinogenesis, we first analyzed the correlation of SOX30 expression with DSP, JUP and DSC3 in lung tissues of SOX30 knockout mice by qRT-PCR, WB and IHC analyses. Consistently, the expression levels of DSP, JUP and DSC3 were significantly decreased in lung tissues of SOX30-knockout mice. (Fig. 7a–b and Additional file 3: Figure S2).

Next, we compared the lung carcinogenesis in *W*/W (wild type of SOX30) and K/K (homozygous deletion of SOX30) mice treated with urethane, an environmental carcinogen that induces ADC in C57BL/6 J mice (data not shown) [29]. H&E staining showed that the number and size of lung tumors were enhanced in K/K mice compared with W/W mice (Fig. 7c). The results suggested that SOX30 plays a vital role in chemical carcinogenesis. To further evaluate whether the promotion of lung tumorigenesis in K/K mice was related to the downregulation of the desmosomal gene expression with activation of the Wnt and ERK pathway, the protein level of SOX30, DSP, JUP, DSC3, CCND1, MMP7, ERK1/2 and p-ERK1/2 were determined by WB in the lung tumor tissues. The protein expression of desmosomal genes were significantly decreased, and CCND1, MMP7, ERK1/2 and p-ERK1/2 were increased in lung tumor tissues of K/K mice compared to W/W mice (Fig. 7d).

## Discussion

Desmosomes are intercellular junctions that tether intermediate filaments to the plasma membrane and represent the major adhesive cell junctions of epithelial cells [30]. It has been reported that desmosome family members play a crucial role in tumor growth and metastasis [21, 31, 32]. Many studies suggest that the expressions of desmosomal genes can be regulated by transcription factors. For instance, SIP1/ZEB2 induces EMT partly by repressing the expressions of DSP and PKP2 [33]. DSG4 is repressed by HOXC13, LEF1 and FOXN1 in hair shaft differentiation [34]. The p53 induced expression of DSC3 implicated in human lung cancer [28]. Stat3 regulates DSG3 transcription in epithelial keratinocytes [35]. KLF5 mediates the transcription of DSG2 in mouse colons [36]. All these data indicated that transcription factors may be important in the regulation of desmosomal genes. Nevertheless, prior to this study, the regulatory mechanisms of desmosomal genes in tumorigenesis remained largely uninvestigated.

In this study, we found differences in expression of desmosmal genes between ADC and SCC. These result indicate that the regulatory mechanisms of desmosomal genes between ADC and SCC are different. Previously, we identified SOX30 as an epigenetically silenced tumor suppressor, inhibiting cell proliferation and inducing cell apoptosis in ADC but not in SCC. Thus, we speculated that the expressions of desmosomal genes might be regulated by SOX30 in ADC. To further explore the regulatory mechanisms of desmosomal genes in ADC and SCC, we analyzed the correlation between desmosomal gene expression and SOX30 expression in ADC and SCC using TCGA data. We found a positive correlation between desmosomal genes and SOX30 in ADC but not in SCC. Further studies demonstrated that SOX30 upregulated the expressions of desmosomal genes by directly binding to the ACAAT motif of their promoter regions in ADC cells, but loss of regulatory function in SCC cells. Because SOX30 acts as a transcription factor, we suspected that there is another molecule necessary for regulatory function of SOX30, and this molecule interferes the function of SOX30 in SCC. Here, we found other desmosomal genes, including DSC1, DSG1, DSC2, PKP1 and DSG3, were also up-regulated by SOX30 (Fig. 2c). Moreover, according to previous reports, PKP3 is dysregulated in lung cancer [11]. Therefore, we analyzed the correlation between SOX30 and PKP3 and found that there is no correlation between the two (Additional file 4: Figure S3a). The qRT-PCR results confirmed that PKP3 expression is not upregulated by SOX30 overexpression (Additional file 4: Figure S3b). We then analyzed the promoter regions of these desmosomal genes and found all of them contain an ACAAT motif except PKP3 (data not shown), indicating that SOX30 upregulated their expression possibly by binding to their promoter regions and activating transcription. Notably, this hypothesis should be empirically tested, and further studies are required to address this aim.

To define whether desmosomal genes are involved in the antitumor effects of SOX30, we focused on the most differentially expressed desmosomal genes associated with SOX30 and chose the three prominent tumor suppressors DSP, JUP and DSC3. These three desmosomal genes were the main targets through which SOX30 suppresses the tumorigenesis and metastasis of ADC. In vitro and in vivo assays demonstrated that desmosomal genes, DSP, JUP and DSC3 were required for SOX30-medicated cell proliferation and metastasis in ADC. Taken together, these results suggested that the “SOX30-desmosomal genes axis” may act as tumor suppressor in ADC. To obtain further insight into the downstream signaling pathway of SOX30-desmosomal genes axis in inhibiting tumor growth and metastasis of ADC, we detected the change of Wnt and ERK signaling pathway related molecules by WB assays and demonstrated that SOX30 suppressed Wnt and ERK signal in a desmosomal gene dependent manner.

To further evaluate the role of the SOX30-desmosomal gene axis in lung tumor development, the chemical carcinogenesis model of SOX30-knockout mice was employed. We found that knockout of SOX30 promotes lung tumor formation in mice with urethane treatment and is associated with desmosomal gene loss. These results suggested that SOX30-desmosomal gene axis might be involved in the occurrence and development of lung tumor. To evaluate this hypothesis, we plan to establish a SOX30 conditional knockout mice to further clarify the important role of the “SOX30-desmosomal gene axis”.

## Conclusions

In conclusion, our results are the first to strongly indicate a key role of SOX30 in regulating most of the desmosomal genes. SOX30 has pivotal roles in ADC cell proliferation and metastasis in vitro and in vivo through the Wnt signal and ERK signal by directly promoting transcriptional activating of desmosomal gene expression. These results provide potential novel mechanisms for the regulation of desmosome genes and new mechanistic insight into the molecular pathogenesis of SOX30-mediated ADC growth and metastasis.

## Additional files

Additional file 1: **Table S1.** Primers used in this study. **Table S2.** The statistical analysis between SOX30 and desmosomal genes. (DOCX 19 kb)

Additional file 2: **Figure S1.** SOX30 upregulate the expression of desmosomal gene in ADC cells but not in SCC cells. a The protein levels of DSP, and JUP and DSC3 were monitored by IF after SOX30 overexpression in A549 and LTEP-a-2 cells. b The protein levels of DSP, and JUP and DSC3 were monitored by IF after SOX30 overexpression in H520 and H226 cells. Scale bar represents 30 mm. (TIF 1116 kb)

Additional file 3: **Figure S2.** The expression of DSP, JUP and DSC3 positively correlates with SOX30 expression in lung tissues of mice. (TIF 3837 kb)

Additional file 4: **Figure S3.** The expression of PKP3 is not not associate with SOX30 expression in ADC. a Heatmaps for correlations between SOX30 and PKP3 in the TCGA lung adenocarcinoma RNAseq (IlluminaHiSeq; *n* = 571) and TCGA lung squamous carcinoma RNAseq (IlluminaHiSeq; *n* = 553) data set. Correlation coefficient R and *P*-values were calculated by a Spearman correlation analysis. b qRT-PCR analysis of PKP3 expression in A549, LTEP-a-2, H520 and H226 cells transiently transfected with the vector control or SOX30 expression vector. ACTIN was used as an internal control. (TIF 826 kb)

## Abbreviations

ADC: Lung adenocarcinoma
ChIP: Chromatin Immunoprecipitation
DSC1: Desmocollin 1
DSC3: Desmocollin 3
DSG2: Desmoglein 2
DSG3: Desmoglein 3
DSP: Desmoplakin
IF: Immunofluorescence cell staining
IHC: Immunohistochemistry
JUP: Junction plakoglobin
NSCLC: Non-small cell lung cancer
PKP1: Plakophilin 1
PKP3: Plakophilin 3
SCC: Lung squamous carcinoma
SOX30: SRY-related HMG-box 30
WB: Western Blot
