# Supplementary material for: SOX30 is a key regulator of desmosomal gene suppressing tumor growth and metastasis in lung adenocarcinoma
Source: J Exp Clin Cancer Res. 2018 May 31;37:111. doi: 10.1186/s13046-018-0778-3 (PMC5984358; doi:10.1186/s13046-018-0778-3)
Supplement: Supplementary file 1 — Table S1. Primers used in this study. Table S2. The statistical analysis between SOX30 and desmosomal genes. (DOCX 19 kb) [file 13046_2018_778_MOESM1_ESM.docx]

Table S1. Primers used in this study

| Description | Species | Name | Sequence |
| --- | --- | --- | --- |
| Primers for | human | DSC1-F | TGATGGAGAACTCGTTGGGTC |
| qRT-PCR/RT-PCR | human | DSC1-R | CCTGGCCCACTTATGGAATAAAA |
|  | human | DSG1-F | GCAGAAACGTGAATGGATCAAGT |
|  | human | DSG1-R | AATTTTGGCGATTGGGTTCCT |
|  | human | DSC2-F | ACACGGCCCAAAACTATACCA |
|  | human | DSC2-R | TTTCCAGTGTCTCTCTCCACATA |
|  | human | JUP-F | CTCTGTGCGTCTCAACTATGG |
|  | human | JUP-R | GATCAAGCCGATGGTTGCCT |
|  | human | DSP-F | GCAGGATGTACTATTCTCGGC |
|  | human | DSP-R | CCTGGATGGTGTTCTGGTTCT |
|  | human | PKP1-F | TTTGCCGTCGGACCAAAAGAT |
|  | human | PKP1-R | GAACCTCGATTGGAGTGGCTC |
|  | human | DSC3-F | GACCCTCGTGATCTTCAGTCG |
|  | human | DSC3-R | TCACTTGACCGGATGAGGTCT |
|  | human | DSG3-F | GCAAAAACGTGAATGGGTGAAA |
|  | human | DSG3-R | TCCAGAGATTCGGTAGGTGATT |
|  | human | PKP3-F | CCCTCAGCAGTCAAGTACCTC |
|  | human | PKP3-R | GCTGCATCGCTGTAGCACT |
|  | human | ACTIN-F | CCACGAAACTACCTTCAACTCC |
|  | human | ACTIN-R | GTGATCTCCTTCTGCATCCTGT |
|  | human | β2m-F | GCTGTCTCCATGTTTGATGTATCTG |
|  | human | β2m-R | GCACGCTTAACTATCTTAACAAGCTTTG |
|  | mouse | DSP-F | GCTGAAGAACACTCTAGCCCA |
|  | mouse | DSP-R | ACTGCTGTTTCCTCTGAGACA |
|  | mouse | JUP-F | TGGCAACAGACATACACCTACG |
|  | mouse | JUP-R | GGTGGTAGTCTTCTTGAGTGTG |
|  | mouse | DSC3-F | AGTTTGAAAGAGTGTCTCAGCTC |
|  | mouse | DSC3-R | ACAACAGCTCTGGTCGGATAA |
|  | mouse | ACTIN | GGAGATTACTGCTCTGGCTCCTA |
|  | mouse | ACTIN | GACTCATCGTACTCCTGCTTGCTG |
|  | mouse | β2m-F | AGGCTTCTCTTTTTCTCCTCTGCTG |
|  | mouse | β2m-R | TTTTCTCTCGACTTCGGTTGGATC |
| Primers for | human | pDSP-F | TTGTTTGGAAAGATGAAAAATTTA |
| ChIP-PCR Analysis | human | pDSP-R | CCAGGAACCGAACCGGGTTTGGAT |
|  | human | pJUP-F | TGATGGTCCTTCAAATGAGA |
|  | human | pJUP-R | TAGAAATTTAAATTAACGGT |
|  | human | pDSC3-F | TGCAAACTCACCAAAAGTGACA |
|  | human | pDSC3-R | GGTTACATGCAATGGCTGGG |

Table S2. The statistical analysis between SOX30 and desmosomal genes

| Gene name | SOX30 | | | | | | | |
| --- | --- | --- | --- | --- | --- | --- | --- | --- |
|  | A549 | | LTEP-a-2 | | H520 | | H226 | |
|  | Fold change | P | Fold change | P | Fold change | P | Fold change | P |
| DSP  JUP  DSC3 | 1.41  1.37  1.86 | 0.002  0.001  0.002 | 5.68  6.68  2.13 | <0.001  <0.001  <0.001 | 1.09  0.95  1.12 | 0.100  0.547  0.060 | 1.07  1.12  0.87 | 0.240  0.063  0.072 |
