# Supplementary figures and images for: SOX30 is a key regulator of desmosomal gene suppressing tumor growth and metastasis in lung adenocarcinoma
Source: J Exp Clin Cancer Res. 2018 May 31;37:111. doi: 10.1186/s13046-018-0778-3 (PMC5984358; doi:10.1186/s13046-018-0778-3)

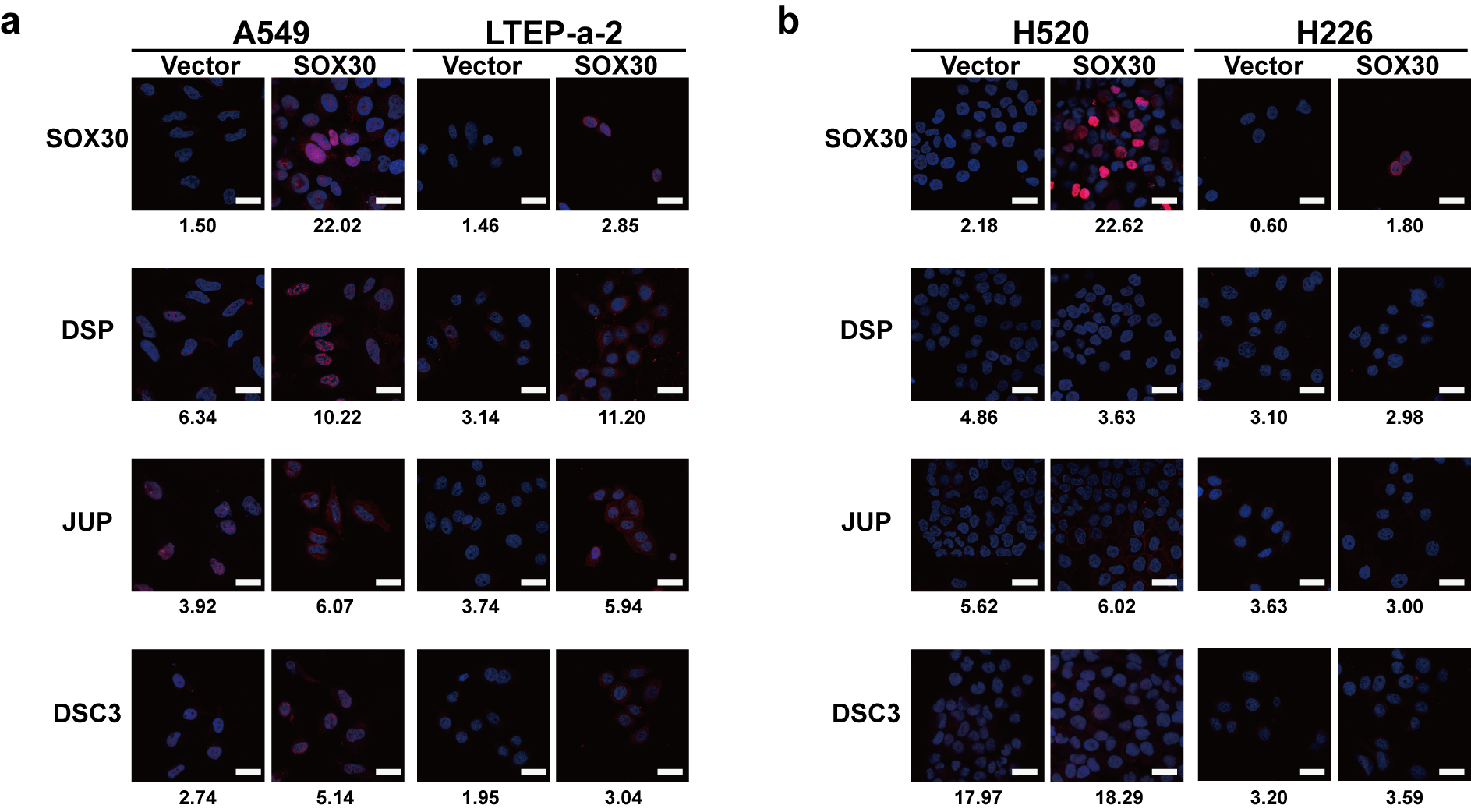

Supplement: Supplementary file 2 — Figure S1. SOX30 upregulate the expression of desmosomal gene in ADC cells but not in SCC cells. a The protein levels of DSP, and JUP and DSC3 were monitored by IF after SOX30 overexpression in A549 and LTEP-a-2 cells. b The protein levels of DSP, and JUP and DSC3 were monitored by IF after SOX30 overexpression in H520 and H226 cells. Scale bar represents 30 mm. (TIF 1116 kb) [file 13046_2018_778_MOESM2_ESM.tif]

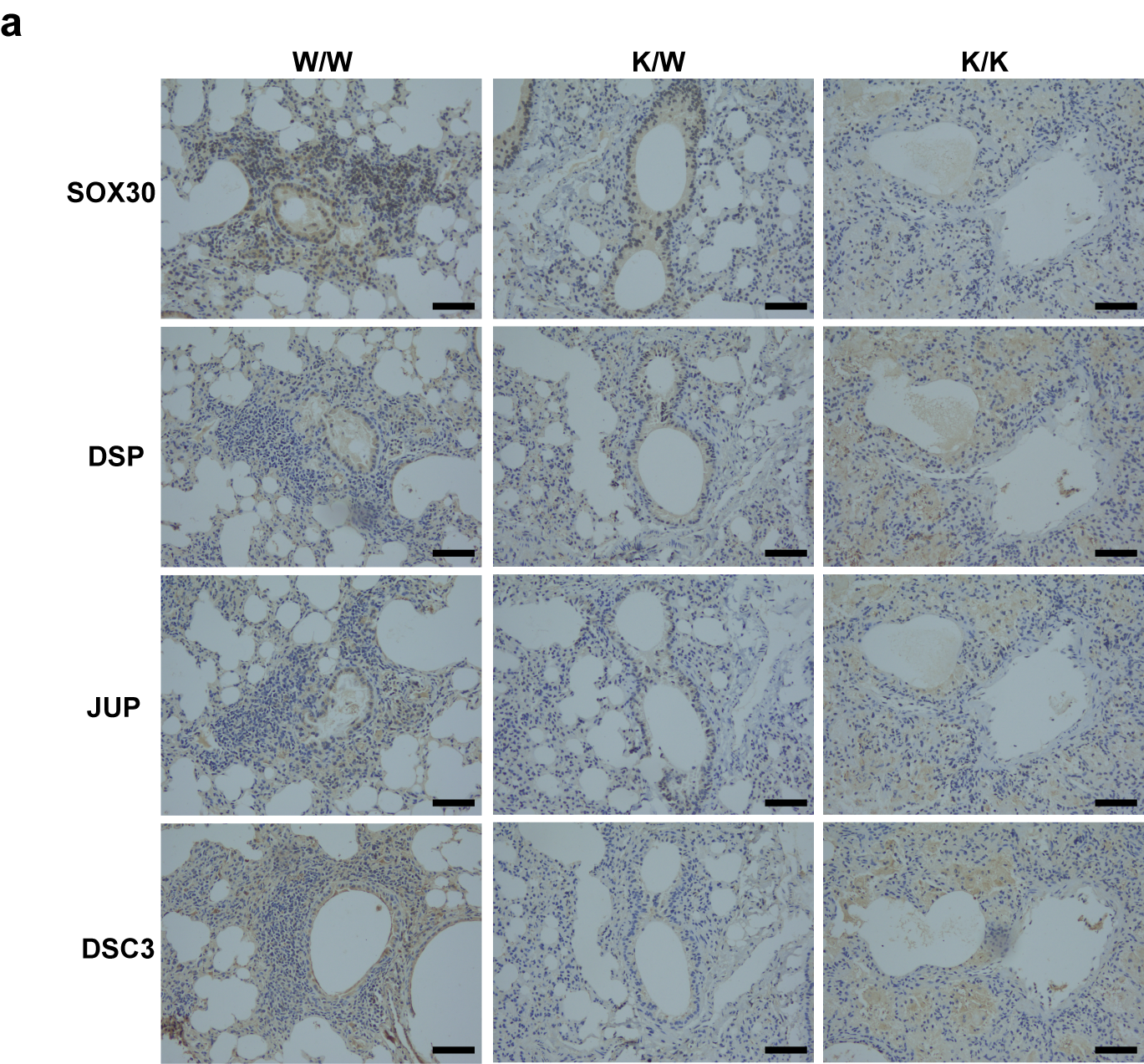

Supplement: Supplementary file 3 — Figure S2. The expression of DSP, JUP and DSC3 positively correlates with SOX30 expression in lung tissues of mice. (TIF 3837 kb) [file 13046_2018_778_MOESM3_ESM.tif]

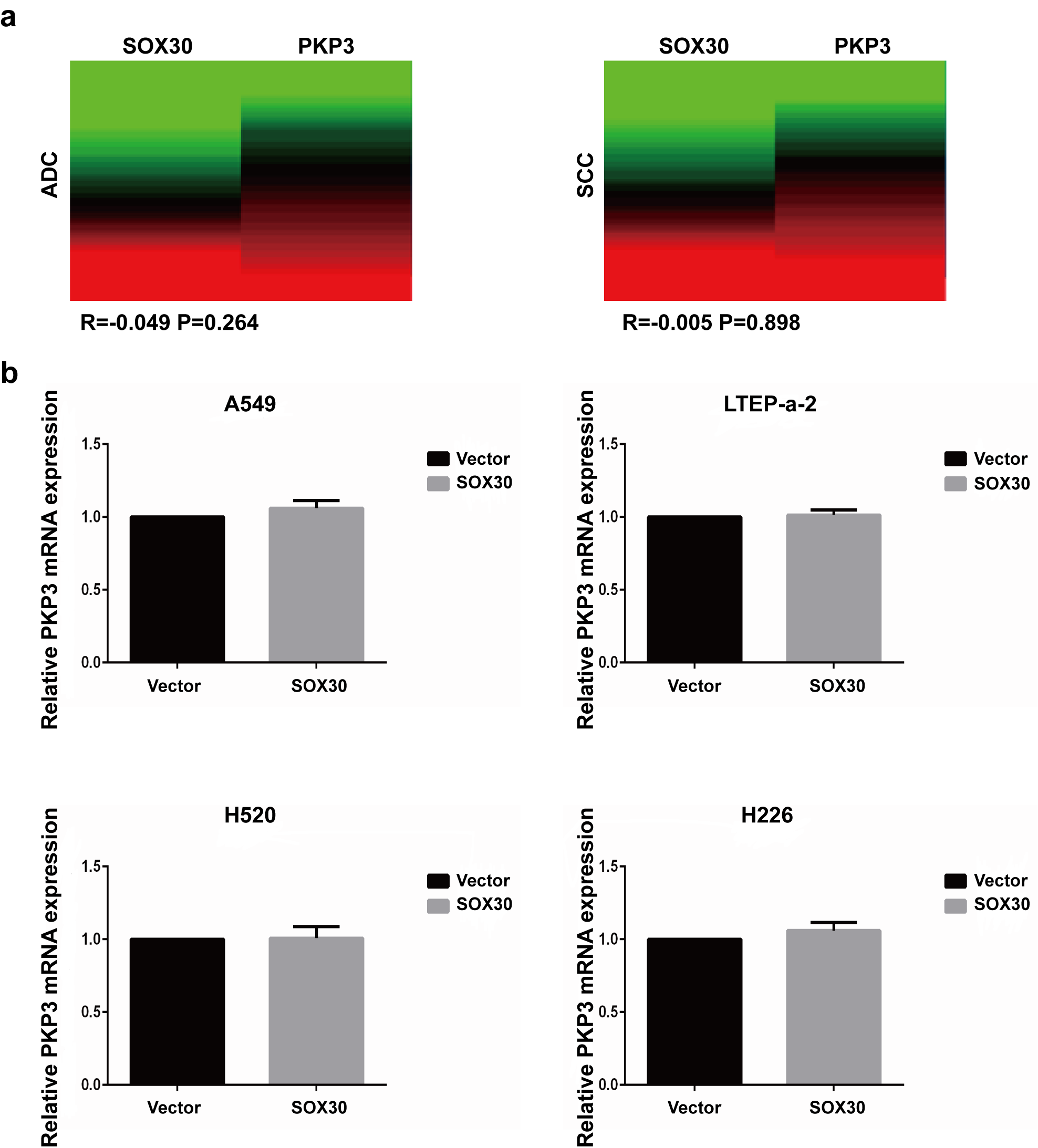

Supplement: Supplementary file 4 — Figure S3. The expression of PKP3 is not not associate with SOX30 expression in ADC. a Heatmaps for correlations between SOX30 and PKP3 in the TCGA lung adenocarcinoma RNAseq (IlluminaHiSeq; n = 571) and TCGA lung squamous carcinoma RNAseq (IlluminaHiSeq; n = 553) data set. Correlation coefficient R and P-values were calculated by a Spearman correlation analysis. b qRT-PCR analysis of PKP3 expression in A549, LTEP-a-2, H520 and H226 cells transiently transfected with the vector control or SOX30 expression vector. ACTIN was used as an internal control. (TIF 826 kb) [file 13046_2018_778_MOESM4_ESM.tif]
